# Raised Intracranial Pressure Syndrome: A Stepwise Approach

**DOI:** 10.5005/jp-journals-10071-23190

**Published:** 2019-06

**Authors:** Swagata Tripathy, Suma Rabab Ahmad

**Affiliations:** 1,2 Department of Anesthesia and Intensive Care, All India Institute of Medical Sciences, Bhubaneswar, Odisha, India

**Keywords:** Complications, Cerebrospinal fluid, Hypertonic saline, Intracranial pressure, Management, Steroids

## Abstract

Raised intracranial pressure (rICP) syndrome is seen in various pathologies. Appropriate and systematic management is important for favourable patient outcome. This review describes the stepwise approach to control the raised ICP in a tiered manner, with increasing aggressiveness. The role of ICP measurement in the assessment of cerebral autoregulation and individualised management is discussed. Although a large amount of research has been undertaken for the management of raised ICP, there still remain unanswered questions. This review tries to put together the best evidence in a succinct manner.

**How to cite this article:**

Tripathy S, Ahmad SR. Raised Intracranial Pressure Syndrome: A Stepwise Approach. Indian J Crit Care Med 2019;23(Suppl 2):S129–S135.

## INTRODUCTION

Raised intracranial pressure (rICP) syndrome is a constellation of clinical symptoms and signs associated with a rise in intracranial pressure. Various pathologies may lead to a rise in intracranial pressure (ICP). The realm of management of raised ICP has progressed over time with the development of new monitoring technology and treatment modalities. There is more clarity now in the understanding of the management; however, there are still some gaps. Here we attempt to review the systematic approach to management of the rICP syndrome.

### Pathophysiology of Raised ICP Syndrome

**Fig. 1:**
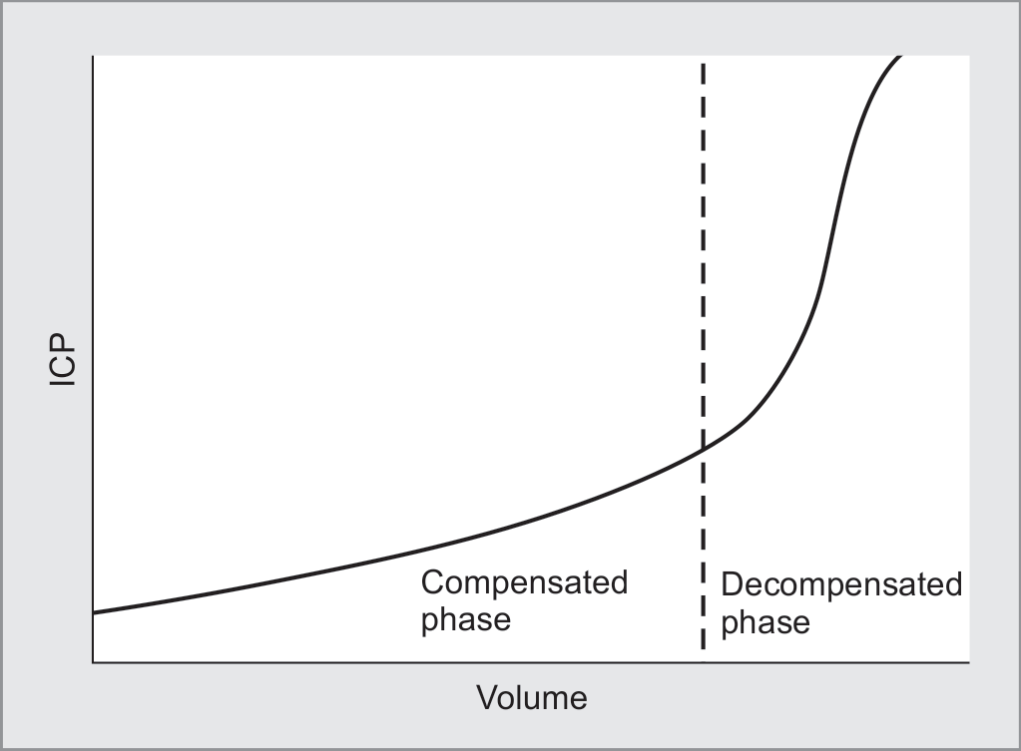
Cerebral volume–pressure curve showing the relationship between ICP and an increase in the intracranial component volume

The Monro-Kellie doctrine originated from the first description of ICP by Scottish anatomist Alexander Monro in 1783.^1^He was supported by his colleague George Kellie some years later. Harvey Cushing, American neurosurgeon, in 1926, formulated the doctrine as we know it today.^2^ He stated that with an intact skull, the volume of the brain, blood, and cerebrospinal fluid (CSF) is constant. An increase in one component will cause a decrease in one or both of the other components. Thus, when there is a growing mass lesion of the brain parenchyma there will be decrease of the CSF or the blood (mainly venous) until the compensatory point is exceeded where we get an elevated ICP^3^ (Fig. 1). Various causes enumerated in Table 1 lead to rICP by increase of either one or all of the three components namely brain, CSF or blood.^4^

Normal ICP is defined as the pressure inside the lateral ventricles or lumbar subarachnoid space in supine position. It normally ranges from <10–15 mm Hg in adults, 3–7 mm Hg in children and 1.5–6 mm Hg in term infants.

We use the Glasgow Coma Scale (GCS) and the Full Outline of UnResponsiveness (FOUR) score to monitor the consciousness of the patient. The FOUR Score was developed for assessing consciousness in intubated trauma patients in whom all the components of GCS cannot be assessed.^5^ It has a good correlation with GCS, shown to give better details of the neurological status in some studies^6^ and can be used in stroke and non-trauma coma also. It is an extensive 17-point scale assessing four domains of neurological function: eye responses, motor responses, brainstem reflexes, and breathing pattern. Any decrease in these scores are associated with worsening consciousness as well as ICP.

**Fig. 2:**
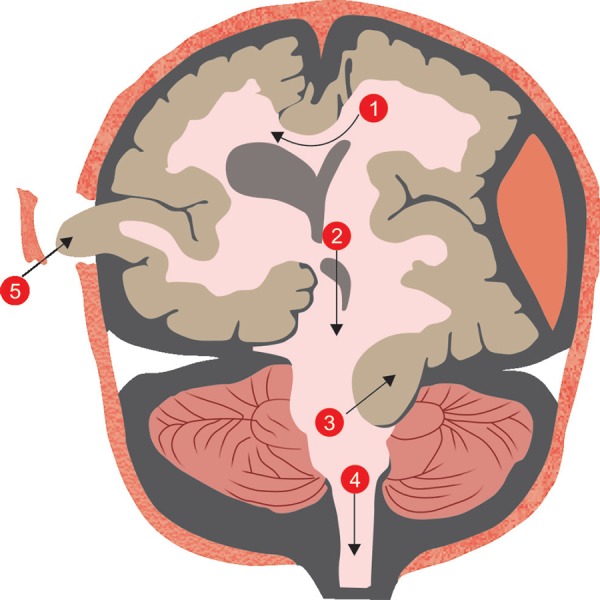
Herniation sites^7^: 1. Subfalcine/Cingulate; 2. Central transtentorial; 3. Lateral transtentorial (Uncal); 4. Tonsillar; 5. Transcalvarial

Clinical clues are the mainstay for deciding the requirement for any imaging or intervention. However, at times these may be missed or appear late (Table 1). These clinical manifestations are a consequence of two major derangements. Firstly rICP poses a danger to the patient in terms of decreased cerebral perfusion pressure (CPP) and the resultant tissue ischemia. Secondly the lesion itself can cause a shift in brain parenchyma manifested as cerebral herniation syndromes (Fig. 2) causing irreversible brain damage and even death (Table 2).

**Table 1:** Causes of raised intracranial pressure

*Pathophysiology*	*Causes*
Focal brain oedema (localized mass lesion)	Traumatic hematomas (extradural, subdural, intracerebral) Neoplasms (gliomas, meningiomas, metastasis) Ischemic or hemorrhagic stroke, abscess
Diffuse brain oedema	Encephalitis, meningitis, diffuse head injury, seizures, encephalopathy (hepatic, toxic, uremic or septic), hypoxemic ischemic encephalopathy, water intoxication, Reye's syndrome
Disturbance of CSF circulation	Obstructive hydrocephalus Communicating hydrocephalus Subarachnoid hemorrhage
Obstruction to major venous sinuses	Depressed fractures overlying major venous sinuses. Cerebral venous thrombosis
Vascular malformations	Arteriovenous malformation
Idiopathic	Benign intracranial hypertension

**Table 2:** Symptoms and signs of rICP

Headache	
Nausea and vomiting	
Systolic hypertension	
Bradycardia	Cushing's triad
Irregular respiration, Cheyne-stokes respiration	
Decreased mental abilities	
Confusion	
Double vision	
Pupils not reacting to light and unequal pupils	
Loss of consciousness and finally coma as the pressure worsens.	

### Management of raised ICP

Increased ICP aggravates secondary brain injury. Secondary brain injury occurs within hours to days after the primary injury. The detrimental processes are cerebral ischemia, cerebral edema and neurochemical interplay of excitatory neurotransmitters, free radicals’ formation, and increased levels of calcium and potassium intracellularly.^8^ The various treatment strategies for lowering ICP should be started along with the treatment of the primary cause. Secondary brain injury is worsened with hypoxia and hypotension. Most commonly the interventions are aimed at decreasing the cerebral blood volume and the fluid component of the brain tissue. Drainage of CSF or surgical removal of brain tissue are done in selected cases.

Most of the evidence for management of rICP has been derived from the published literature on traumatic brain injury (TBI), in particular the Brain Trauma Foundation (BTF) guidelines, fourth edition published in 2017.^9^

## GENERAL MANAGEMENT/TIER ZERO

### Resuscitation: Airway, Breathing and Circulation

#### Airway

Proper and prompt management of airway, breathing and circulation prevents hypoxia, hypercapnia and hypotension. Hypoxia increases ICP by vasodilatation and cerebral edema. Coughing or bucking during laryngoscopy and intubation can cause further increse in the ICP. Hence sedatives should always be used before intubation even if the patient is unresponsive. Esmolol, labetalol and lignocaine can also be used to blunt the hemodynamic responses to laryngoscopy.

#### Ventilation

Hypercarbia is a potent cerebral vasodilator causing increase in cerebral blood volume and ICP. Hence hypoventilation should be avoided and normocapnia maintained.

#### Blood Pressure

Hypotension will decrease the cerebral perfusion pressure in a brain with impaired autoregulation. BTF guidelines recommend maintaining systolic blood pressure at ≥ 100 mm Hg for patients 50 to 69 years old or at ≥ 110 mm Hg or above for patients 15–49 or >70 years old to decrease mortality and improve outcomes (Level III).

Elevated blood pressure is seen commonly in patients with rICP. This is a compensatory mechanism to maintain the CPP and it is unwise to control it. However, when autoregulation is impaired, as in traumatic brain injury (TBI) this might increase the cerebral blood flow (CBF) and ICP. It is also carries a risk of causing intracranial hemorrhage in certain conditions like hemorrhagic stroke or in the postoperative neurosurgery patient. Systemic hypertension usually resolves with sedation. For treatment antihypertensive drugs such as sympatholytics: β-blockers (labetalol, esmolol) or centrally acting α -agonists (clonidine) can be used. Vasodilating drugs, such as nitroprusside, and nitro-glycerine should be avoided as these increase ICP.

**Table 3:** Comparison of different sedatives and supporting evidence

*Sedative*	*Advantages*	*Cautions*	*Literature*
Propofol	Recommended for the control of ICP (level IIb)	High dose or prolonged infusion: Propofol infusion syndrome	First line sedative ^15^
Midazolam	Safe in rICP lowest incidence of spreading depolarizations, a potentially modifiable secondary injury mechanism	Tachyphylaxis	First line sedative ^15^ -Robin et al.: iv bolus of midazolam prior to suctioning significant reduction in ICP ^16^
Ketamine	NMDA receptor antagonist neuroprotective effect inconclusive Used as an adjunct with other sedatives: has shown to decrease ICP ^17^	Early case reports concluded that it increases CSF secretion increases ICP	Intraoperative administration of ketamine for craniotomy: 1 mg/kg Ketamine reduced ICP ^18^ Further RCTs required
Barbiturates	Only for elevated ICP refractory to maximum standard medical and surgical treatment.	Prophylactic use against the development of raised ICP: not recommended. Hemodynamic stability is essential	Brain Trauma Foundation guidelines^9^
Dexmedetomidine	Sedation and analgesia without respiratory depression Patient is arousable, facilitates neurological assessment Rapid distribution and elimination properties	Hypotension, bradycardia, Agitation Further studies to establish its dose and duration	Aryan et al., neurosurgical patients: safe and effective, mean ICP decreased.^19^ avoid loading dose, higher maintenance doses to ensure adequate sedation
Inhaled sedatives: sevoflurane and isoflurane	Emerging as sedative agent	Flow metabolism uncoupling action Acute cerebrovascular disease: sevoflurane was associated with a significant increase in ICP ^20^	Subarachnoid hemorrhage without rICP: 0.8 % isoflurane significantly improved regional CBF with modest effect on ICP when compared with propofol ^21^

*NMDA: N-methyl-D-aspartate

#### CPP

BTF recommends target CPP value for survival and favourable outcomes between 60 mm Hg and 70 mm Hg, depending on the autoregulatory status of the patient. Values above 70 carry a risk of acute respiratory distress syndrome^9^ (Level III).

### Fluids

A subgroup analysis of patients with TBI in the Saline vs. Albumin Fluid Evaluation (SAFE) study found that mortality was higher in patients resuscitated with albumin compared with saline, but the mechanism was unknown.^10^ Later it was propounded that the most likely mechanism of increased mortality was the rICP during the use of albumin in the first week.^11^ Hypo-osmolar fluids should be avoided. Hyponatremia should be corrected since it increases cerebral edema.

### Sedation and Analgesia

Sedation and analgesia in patients with rICP prevent coughing, bucking and agitation, facilitates mechanical ventilation and suctioning as well as enables seizure control. It exerts cerebral protective effects primarily by reducing cerebral metabolic rate of oxygen consumption (CMRO_2_) and CBF which is tightly coupled to CMRO_2_.^12^

Minimal periods of sedation interruption in patients with rICP will prevent ICP spikes.^13^ There is no evidence that one sedative agent is more efficacious than another for improvement of ICP (Table 3).^14^

Sedatives should be adequately supplemented with analgesics. Newer opioids like fentanyl are the primary analgesics. Large bolus doses of opioids, however, have potentially deleterious effects on ICP and CPP.^14^ Non-opioid analgesics help to minimize opioid use. Rarely, ICP control necessitates the use of neuromuscular blockade.

### Facilitation of Cerebral Venous Drainage

Head end of the bed should be kept elevated at 15–30 degrees with the head in a neutral position to enhance cerebral venous drainage and to promote the circulation of CSF from intracranial to spinal compartment. Any tight circumferential tracheostomy or endotracheal tube ties or cervical collar may need adjustment to prevent internal jugular vein compression. Any rise in intrathoracic or intra-abdominal pressures can also interfere with venous drainage.

### Fever Control

Fever increases metabolic rate by 10% to 13% per degree Celsius and is a potent vasodilator. It increases ICP. Fever should be controlled by antipyretics and hydrotherapy.

### Glucocorticoids

Neurological deficit secondary to vasogenic oedema due to brain tumors, abcesses or non-infectious neuroinflammations responds well to steroid use as a temporising intervention. rICP, when present decreases over the following 2–5 days. Intravenous dexamethasone is commonly used, at a dose of 4 mg every 6 hours.

Steroids are contraindicated in treating raised ICP or for improving outcome secondary to TBI or spontaneous hemorrhage **(**Level I Evidence)^7^. Use of methylprednisolone for 48 hours in CRASH trial resulted in a significant increase in the risk of death.

A non-contrast CT scan head should be performed when patient can be transported safely after Tier 0 management.^22^

### Tier 1 Specific Therapy

#### Osmotic Therapy

Hyperosmolar therapy has been regarded as the mainstay of treatment of raised ICP. Hyperosmolar agents help to decrease ICP by effectively reducing brain water. It can be traced back to the publication of Weed and McKibben.^23^ However a Class I evidence is still lacking.

Though mannitol is a time-tested agent, more recently, hypertonic saline (HTS) formulations, have been investigated. In 1988 Worthley et al. first found that HTS reduced rICP which was refractory to mannitol.^24^ However, for acute elevations in ICP, either of the hyperosmolar therapy has shown equal efficacy in lowering ICP. For refractory intracranial hypertension, hypertonic saline may be preferred as concluded by a recent meta-analysis by Gu et al.^25^

#### Mannitol

Mannitol is effective for control of raised ICP at doses of 0.25–1 g/kg body weight. Doses > 200g/day may cause acute renal failure (ARF). The serum osmolarity should be monitored and kept below 320 mOsm/kg. A more reliable marker of serum mannitol level may be the osmolar gap (OG).

Mannitol lowers the ICP 1–5 minutes after intravenous administration, and its peak effect is at 40 minutes. The duration of effect is 90 minutes – 6 hours. Mannitol will cause an initial plasma expansion that will increase CBF. A damaged blood-brain barrier (BBB) may worsen vasogenic oedema, however.

Mannitol is associated with many other complications. Hypotension with rapid administration (< 5min), rebound increase in ICP, volume overload and electrolyte imbalances (hypo/ hypernatremia) and an early but transient decrease of serum bicarbonate and increases in serum potassium. Arterial hypotension (systolic blood pressure < 90 mm Hg) should be avoided.^26^ Mannitol use should be restricted prior to ICP monitoring in patients with signs of transtentorial herniation.^26^

Mannitol can be administered via a peripheral vein. An osmolar gap of 20 mmol/dl marks inadequate clearance of mannitol and will increase the risk of rebound rise in ICP. Mannitol may need to be warmed to dissolve crystals which precipitate in the bottle.

#### Hypertonic saline (HTS)

Gaining popularity,^27^ it is available concentrations ranging from 3% to 23.4%. It remains within the vascular compartment longer than mannitol and so is useful in treating the hypovolemic patient. It has a better reflection coefficient than mannitol (agents with lower reflection coefficients have a greater risk of accumulating inside the brain) and tends to cross the BBB less. HTS can also be used to treat hyponatremia, which untreated can worsen brain oedema.

*Dose*: Bolus dosing 3%: 2.5–5 mL/kg over 5–20 minutes, 5%: 2.5–5 mL/kg over 5–20 minutes, 7.5%: 1.5–2.5 mL/kg over 5–20 minutes, 23.4%: 30 mL over 10–20 minutes. Hypertonic saline may be given in continuous infusion. Serum sodium beyond 160 mEq/dL is unlikely to provide any further benefit.

Central line access is recommended. Frequent serum sodium levels will need to be monitored prior to the next scheduled dose to prevent osmotic demyelination due too rapid rise of serum sodium if the patient had hyponatremia to begin with . Duration of effect is 90 min to 4 hrs. Adverse effects include thrombophlebitis, coagulation abnormality and hyperchloremic metabolic acidosis.

If ICP is controlled with Tier 1 measures then consider repeating CT scan to rule out any new processes.

#### Tier 2

If ICP is not controlled with Tier 1 medical interventions, decompressive surgical options should be considered. If patient is not fit for surgery then other Tier 2 interventions should be applied. Sedation depth can be increased by using agents like propofol.^22^

### Resection of Mass Lesions

These are done to decrease the ICP and as a definitive therapy for the lesions. Abscesses must be drained; acute epidural and subdural hematoma must be evacuated. Resection of intracerebral lesion (lobar/cerebellar hemorrhage) or brain parenchyma (eg: contusion) also aid in decompression. Tension pneumo-encephalous should be tapped.

### Cerebrospinal Fluid (CSF) Drainage

CSF drainage by external ventricular drain (EVD) lowers ICP immediately by reducing intracranial volume. In a diffusely swollen brain, sudden decompression may cause the ventricles to collapse. Continuous drainage of CSF by an EVD zeroed at the level of the midbrain (5–10 cm above the external auditory meatus) may be more beneficial in reducing the ICP than intermittent drainage.^9^ (Level III) Use of CSF drainage to lower ICP in patients with an initial GCS <6 during the first 12 hours after TBI may be considered in the absence of coagulopathy.

### Decompressive Craniectomy (DC)

DC involves removal of a portion of the skull vault resulting in immediate decrease of the ICP. It is undertaken in patients with diffuse cerebral swelling due to TBI, meningoencephalitis, stroke with brain edema and non-infectious neuro-inflammatory conditions (eg: acute demyelinating encephalopathy). Usually a decompressive hemicraniotomy (DHC) is done.

Reported complications of decompressive craniectomy include hydrocephalus, hemorrhagic swelling ipsilateral to the craniectomy site, and subdural hygroma. A large frontotemporoparietal DC (not less than 12 x 15 cm or 15 cm diameter) is recommended over a small frontotemporoparietal DC for reduced mortality and improved neurologic outcomes in patients with severe TBI.^9^

Although there is evidence of decrease in ICP with DC, several studies have been performed to assess whether the benefits can be achieved in terms of good functional outcome or decrease in mortality.

Cerebral oedema after massive malignant middle cerebral-artery infarction (mMCAi) results in increased ICP. It often has devastating consequences leading to brain herniation and death. A recent meta-analysis which included most large RCTs to date (Table 4) concluded that DC results in large reductions in mortality (RR 2.05, 95%CI 1.54–2.72; *p* < 0.00001). Surgery improved the likelihood to survive with a mRS 0–3 (RR 1.58, 95%CI 1.02–2.46;*p* = 0.04).^28^ As the DESTINY II trial included elderly patients, age can not be a criteria for exclusion of patients, although the functional outcomes of this population is arguably worse than that of younger patients.

**Table 4:** Trials assessing the effect of decompressive craniectomy in massive malignant middle cerebral-artery infarction

*Sl. No.*	*Trials*	*Year*	*Conclusion*
1	DECIMAL: Decompressive craniectomy in malignant MCA infarcts^29^	2007	Absolute mortality reduction of 52% with DC, No significant difference in functional outcomes.
2	DESTINY I: Decompressive surgery for the treatment of malignant infarction of the MCA ^30^	2007	Mortality reduction from 88% to 47% with DC after 1 month.
3	HAMLET: Hemicraniectomy after MCA infarction with life-threatening edema trial ^31^	2009	ARR 38% for fatality, but no difference in functional outcomes.
4	HeADDFIRST: Hemicraniectomy and Durotomy Upon Deterioration From Infarction-Related Swelling Trial^32^	2014	Difference in mortality was not significant
5	DESTINY II: Decompressive surgery for the treatment of malignant infarction of the MCA in elderly patients >60 years age^33^	2014	Significant reduction of severe disability.

**Table 5:** Evidence for the effect of decompressive craniectomy in patients with post-traumatic refractory intracranial hypertension

*Sl. No.*	*Trials*	*Conclusion*
1.	DECRA study Limited by its restrictive eligibility criteria ^34^	No benefit in terms of functional outcome at 6 months from bifrontal DC
2.	RESCUE ICP: ^35^ Randomised Evaluation of Surgery with Craniectomy for Uncontrollable Elevation of ICP broader range of patients: more typical of those encountered in routine practice published after BTF-IVth edition	Reduction of mortality by 22% Higher rates of vegetative state, and severe disabilities than medical management Similar rates of moderate disability and good recovery with surgery than medical management.

**Table 6:** Contraindications of hyperventilation^36^

Prophylactic
For first 24 hours of severe TBI when CBF often is reduced critically^9^
For prolonged periods (>4–6 hours)
Without brain oxygenation monitoring
Should not stop suddenly: risk of rebound rICP

The role of DC in patients with post-traumatic intracranial hypertension that is refractory to medical management alone also remains unclear. Bifrontal DC is not recommended to improve outcomes as measured by the GOS-E score at 6 months post-injury in severe TBI with diffuse injury (without mass lesions), and with refractory ICP elevation. However, this procedure has been demonstrated to reduce ICP and ICU days (Level IIA) (Table 5).

### Hyperventilation

Hyperventilation is recommended only as a temporizing measure for the reduction of elevated ICP in the setting of refractory hypertension and for brief periods (<2 hours) in cases of cerebral herniation or acute neurologic deterioration.^9^

The effect of hyperventilation is almost immediate but lasts for only 4–6 hours after which pH of the CSF rapidly equilibrates to the new PaCO_2_ level. As the CSF pH equilibrates, the cerebral arterioles dilate again. A goal of pCO_2_ 30–35 mm Hg should be the target.^22^ Prolonged prophylactic hyperventilation with PaCO_2_ of ≤25 mm Hg is not recommended by BTF (Level IIB) as there is a risk of cerebral ischemia (Table 6). If hyperventilation is used, SjO_2_ (Jugular venous oxygen) or BtpO_2_ (brain tissue oxygen) measurements are ideally recommended to monitor oxygen delivery.

### Antiseizure Therapy

Seizure activity will increase cerebral metabolic rate (CMR) and CBF. CBF in excess of tissue demand leads to increased ICP. The latest BTF guidelines have recommended phenytoin to decrease the incidence of early posttraumatic seizures (PTS), within 7 days of injury. Prophylactic use of phenytoin is not recommended for preventing late PTS. In patients with severe TBI as well as with other causes of coma and rICP, seizures may be nonconvulsive, detected only with electroencephalographic monitoring. There is insufficient evidence to recommend levetiracetam compared with phenytoin regarding efficacy in preventing early PTS and toxicity.

#### Tier 3

They are the most aggressive measure to reduce ICP with most serious adverse effects. Hence used only in refractory cases. Good quality evidence is sparse.

*Barbiturate coma*: Barbiturates reduce ICP. The mechanism of ICP reduction by barbiturates is probably the result of a coupled reduction in CBF and CMR. Pentobarbital is not preferred as it may result in hypotension needing vasopressor support. Thiopentone is given in a loading dose of 5mg/kg over 30 minutes followed by infusion of 1-5 mg/kg hour until the electroencephalogram shows a burst suppression pattern. During administration blood pressure should be monitored as it can cause hypotension. Complications of barbiturate coma include hypotension, hypokalemia, respiratory depression, infections due to immune suppression, and hepatic and renal dysfunction.

### Therapeutic Hypothermia

Hypothermia reduces the CMR in a similar way as pentobarbital coma. It also reduces the basal component of cellular metabolism along with suppression of electrical activity of brain.^37^ The evidence for benefit is stronger for post-cardiac arrest patients and for neonatal hypoxic ischemia. There is a predictable decrease in ICP with the use of moderate hypothermia (target core temperature 32–34°C). There are various adverse effects of hypothermia such as shivering, cardiac arrhythmias, electrolyte disturbances and sepsis. Rewarming should be done slowly to avoid rebound severe intracranial hypertension.

Recently, the large multicenter EUROTHERM trial randomized patients with recent TBI and ICP refractory to tier one strategies, to receive either therapeutic hypothermia for a minimum of 48 hours plus standard care, or standard care alone. They found that the two approaches were equivalent in reducing ICP, but the intervention group paradoxically had a statistically significant increase in the odds of poor functional outcome and mortality at 6 months.^38^ Hence prophylactic hypothermia early (within 2.5 hours) or short-term (48 hours post-injury) is not recommended to improve outcomes in patients with diffuse TBI.

Role of ICP measurement in assessment of cerebral autoregulation:

Patients with deranged brain function may benefit from the use of additional neuromonitoring. Brain tissue hypoxia can occur even when ICP and CPP are normal. The functioning of these monitors is based on the principle of balance of cerebral metabolic demand and supply which is overlooked by ICP and CPP. Similarly, cerebral metabolic by-products as detected by cerebral microdialysis are the indicators of balance of metabolic demand supply and may alter independent of ICP and CPP.^39^ Currently, there are several indices of cerebral autoregulation to suggest autoregulation failure.^40^

In monitoring of cerebral autoregulation, ICP has been used as a surrogate of cerebral blood flow. Correlated with mean arterial pressure changes, it has given rise to various indices such as the pressure reactivity index (PRx), diastolic coefficient index, low frequency autoregulation index etc.^41^ These are used to assess cerebral autoregulation as a part of multimodality monitoring (MMM) to individualize patient care.

**Fig. 3:**
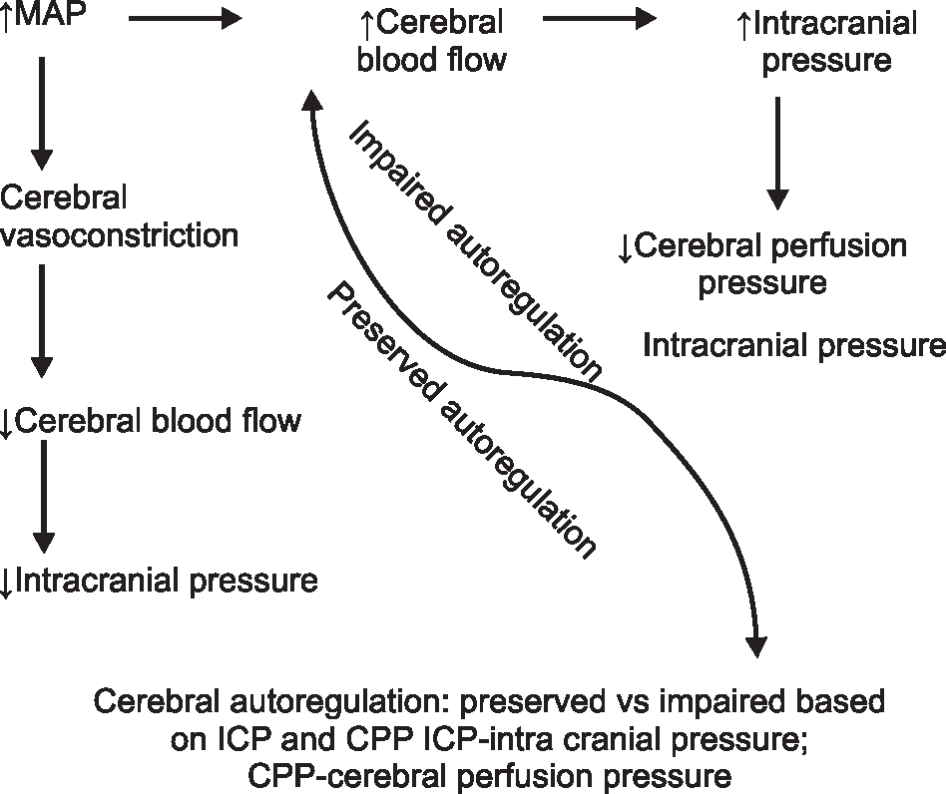
Effect of cerebral autoregulation on intracranial pressure

Mean arterial pressure (MAP) and ICP along with PRx are used in specific centres. Along with feedback from microdialysis and brain oxygenation parameters it should be possible to determine and validate an optimal cerebral perfusion pressure for each unique patient and disease state so that the detrimental effects of pressure changes in a brain with impaired autoregulation may be avoided (Fig. 3).

## CONCLUSION

Timely recognition and management of rICP improves patient outcomes when performed in a step wise manner and increasing aggressiveness. Direct benefits on functional outcomes may be more apparent when the management is done in a more individualized manner. Recent research and attempts are ongoing toward precision care of acute brain injury, of which ICP remains an integral component.
